# Community-Acquired Methicillin-Resistant Staphylococcus aureus Pneumonia

**DOI:** 10.7759/cureus.72166

**Published:** 2024-10-22

**Authors:** Swati Mahapatra, Sukhila Reddy, Shivangini Duggal, Hedaia Algheriani, Monica Botros, Abhinav Vulisha, Sheldon Rao

**Affiliations:** 1 Internal Medicine, Texas Tech University Health Sciences Center El Paso, El Paso, USA; 2 Pulmonology, Texas Tech University Health Sciences Center El Paso, El Paso, USA

**Keywords:** complicated community-acquired pneumonia (ccap), complication of empyema, methicillin-resistant staphylococcus aureus (mrsa), mrsa prevalence, severe community-acquired pneumonia

## Abstract

Community-acquired pneumonia secondary to methicillin-resistant Staphylococcus aureus (MRSA) is a rare occurrence that is known to occur in young adults with no recent hospitalization. It is known to present with hemoptysis and is usually associated with concomitant influenza infection and is classically diagnosed by sputum culture or broncheoalvolar lavage. Routine treatment of community-acquired pneumonia does not suffice in the event of MRSA growth, and, if left untreated, can develop into serious medical complications of sepsis, multiloculated fluid collections, and multiorgan failure. In the southwestern region, particularly between the two predominantly Hispanic cities of El Paso and Las Cruces, there has been an increased incidence of MRSA strains isolated. This case report highlights the importance of identifying MRSA pneumonia in a clinical setting, particularly in southwestern regions.

## Introduction

*Staphylococcus aureus (S. aureus)* is a gram-positive, aerobic, and facultative anaerobic cocci. It is known for being a common component of the skin flora and has classically colonized the nose and hands.* S. aureus* is known for its virulence factors, including toxins, immune-modulatory factors, and exoenzymes [1]. Despite this, *S. aureus* is susceptible to most antibiotics. However, if the methicillin resistance gene is present on the staphylococcal chromosome, the microbe is now resistant to most beta-lactam antibiotics [1]. Methicillin-resistant *S. aureus *(MRSA) has been cited in the literature as most commonly present with nosocomial pneumonia, both hospital and ventilator-acquired. However, MRSA community-acquired pneumonia is rare and associated with high morbidity and mortality [2,3]. Pneumonia accounts for an estimated 50,000 staphylococcal infections per year in the United States alone. In the southwestern region, particularly between the two predominantly Hispanic cities of El Paso and Las Cruces, there has been an increased incidence of MRSA strains isolated [4]. Pneumonia accounts for approximately 50,000 staphylococcal infections a year in the United States. It has been noted clinically that mortality from cases of MRSA pneumonia can exceed 50%, which highlights the severity of the disease that can be attributed to MRSA [5]. Here, we present a case of severe community-acquired pneumonia caused by MRSA in a young adult that resulted in sepsis along with empyema requiring an extensive hospital stay.

## Case presentation

**Figure 1:**
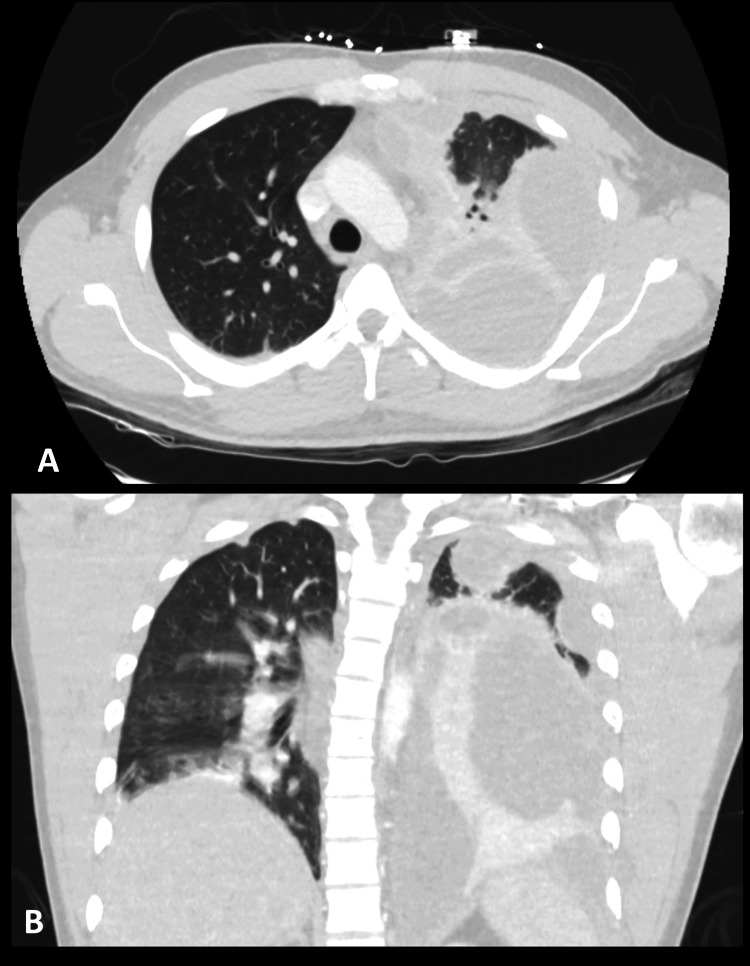
(A) CT angiography of the chest with contrast showing loculated left-sided collections with associated pleural thickening. (B) CT angiography of the chest with contrast in a coronal plane showing loculated left-sided collections with associated pleural thickening. Complete atelectasis of the left lower lobe and lingula can be noted.

A 27-year-old male with no significant medical history was brought to the emergency department for chief complaints of substernal chest pain, abdominal pain, shortness of breath, and fevers for the past four days. His social history was significant for jail time six months ago. The patient had been discharged from prison and presented to the hospital due to continued substernal pain. He was tachycardic, febrile, normotensive, and saturating at 98% on 6 L of oxygen upon admission. His labs on admission revealed leukocytosis with neutrophilia, elevated bilirubin, aspartate transaminase:alanine transaminase ratio of almost 2:1, elevated alkaline phosphatase, elevated lipase, lactic acidosis, and elevated initial troponin (Table 1). The patient was started on ceftriaxone and azithromycin for community-acquired pneumonia and morphine for pain management. Arterial blood gas analysis showed respiratory alkalosis with metabolic alkalosis likely due to persistent tachypnea. Despite treatment and fluid resuscitation, the patient continued to be septic and had increasing oxygen requirements. Treatment was broadened to include anaerobic, MRSA, and pseudomonal coverage. Sputum cultures were obtained due to suspicion for MRSA due to the lack of clinical improvement. His CT of the chest showed multiloculated left pleural fluid collections in addition to pancreatitis which was confirmed on the CT of the abdomen and pelvis (Figure 1). Due to the loculations of the pleural fluid, interventional radiology was consulted, and a chest tube was placed on the left side along with a guided thoracentesis. Pleural fluid analysis showed an extremely elevated pleural fluid lactate dehydrogenase (LDH) compared to the serum LDH that was positive for an exudative effusion. However, due to the diffuse nature of the loculations, the patient was referred to cardiothoracic surgery where he was then taken for a thoracotomy with decortication and multiple chest tube placements. The postoperative culture was unremarkable due to significant antibiotic usage prior; however, pleural fluid cultures grew MRSA. The patient was able to wean off oxygen and was sent home with intravenous (IV) vancomycin to complete two weeks of IV antibiotics post-decortication.

**Table 1:** Laboratory results on admission.

Test	Results	Normal range
White blood count	30.2 × 10^3^/µL	4.50–11 × 10^3^/µL
Hemoglobin	16.8 g/dL	12.0–15.0 g/dL
Platelets	387 × 10^3^/µL	150–450 × 10^3^/µL
Serum sodium	133 mmol/L	135–145 mmol/L
Creatinine	0.9 mg/dL	0.70–1.3 mg/dL
Albumin	4.0 g/dL	3.4–5.4 g/dL
Total bilirubin	5.2 mg/dL	0.1–1.2 mg/dL
Direct bilirubin	2.4 mg/dL	<0.3 mg/dL
Aspartate aminotransferase	67 IU/L	14–20 IU/L
Alanine aminotransferase	40 IU/L	29–33 IU/L
Alkaline phosphatase	193 IU/L	44–147 IU/L
Serum protein	5.6 IU/L	6.3–8.2 g/dL
Serum lactate dehydrogenase	558 IU/L	120,246 IU/L
Pleural lactate dehydrogenase	>8,600 IU/L	<10,000 IU/L
Pleural total protein	4.3 g/dL	<1.5 g/dL

## Discussion

Community-acquired pneumonia caused by MRSA has been found to lead to critical illness and death. Panton-Valentine leukocidin (PVL) is known as a virulence factor of *S. aureus* that is associated with serious complications. Incidences of infections in young adults and healthy individuals with no exposure to healthcare settings and no classical risk factors have emerged [4]. This is seen in the above case as well in which a young healthy adult with no recent hospitalization was found to have MRSA pneumonia. Our patient did have an incarceration history but of six months prior which did not correlate with the onset of symptoms. Nor was he immunocompromised. We would like to emphasize that strains of MRSA have been isolated in the El Paso population which might be a risk factor in the development of MRSA pneumonia in our patient. Clinical features suggestive of MRSA pneumonia include concurrent influenza infection, hemoptysis, multi-lobar infiltrates, and neutropenia [4,6]. Sputum gram stains, cultures, and two sets of blood cultures should be obtained. Typically for MRSA pneumonia, linezolid remains the standard [7]. If vancomycin or teicoplanin are used, one must investigate the PVL status. If positive, combination therapy would be required with clindamycin or rifampicin [7]. Daptomycin was not used as its efficacy in MRSA pneumonia is inhibited by pulmonary surfactant [8]. Clinical suspicion and early treatment with targeted antibiotics are key for successful eradication. Despite aggressive diagnosis and treatment, there remains a high mortality rate associated with MRSA pneumonia [9]. *S. aureus* pneumonia also imparts a significant burden on the economy as the cost for patients is an upward excess of $35,000 [10].

## Conclusions

MRSA pneumonia is known to cause severe pneumonia in young healthy adults and has been linked to an increase in mortality due to disease severity. As discussed in this case report, it can cause a complicated and prolonged healthcare course. Understanding the prevalence, clinical presentation, and proper antibiotic regimen are crucial to better understanding and managing MRSA pneumonia, particularly in communities where the prevalence of virulent strains may be increasing. Due to its high mortality and morbidity, healthcare cost, and serious complications associated with it, it is essential to appropriately detect and treat this infection in a timely manner. Geography may prove to be an additional consideration in the treatment of community-acquired pneumonia, given the increasing prevalence of MRSA strains in certain regions such as Southwest America.
